# Ciprofloxacin Causes Persister Formation by Inducing the TisB toxin in *Escherichia coli*

**DOI:** 10.1371/journal.pbio.1000317

**Published:** 2010-02-23

**Authors:** Tobias Dörr, Marin Vulić, Kim Lewis

**Affiliations:** Antimicrobial Discovery Center, Department of Biology, Northeastern University, Boston, Massachusetts, United States of America; Harvard University, United States of America

## Abstract

Bacteria induce stress responses that protect the cell from lethal factors such as DNA-damaging agents. Bacterial populations also form *persisters*, dormant cells that are highly tolerant to antibiotics and play an important role in recalcitrance of biofilm infections. Stress response and dormancy appear to represent alternative strategies of cell survival. The mechanism of persister formation is unknown, but isolated persisters show increased levels of toxin/antitoxin (TA) transcripts. We have found previously that one or more components of the SOS response induce persister formation after exposure to a DNA-damaging antibiotic. The SOS response induces several TA genes in *Escherichia coli*. Here, we show that a knockout of a particular SOS-TA locus, *tisAB/istR*, had a sharply decreased level of persisters tolerant to ciprofloxacin, an antibiotic that causes DNA damage. Step-wise administration of ciprofloxacin induced persister formation in a *tisAB*-dependent manner, and cells producing TisB toxin were tolerant to multiple antibiotics. TisB is a membrane peptide that was shown to decrease proton motive force and ATP levels, consistent with its role in forming dormant cells. These results suggest that a DNA damage–induced toxin controls production of multidrug tolerant cells and thus provide a model of persister formation.

## Abstract 2

Persisters are specialized survivor cells that arise in populations of *E. coli* after antibiotic-mediated DNA damage induces the production of a small membrane-acting peptide TisB, which causes reversible dormancy. The TisB-dependent persisters are tolerant to multiple antibiotics.

## Author Summary

Bacterial populations contain a small number of dormant cells (*persisters*) that are tolerant to antibiotics. Persisters are not mutants, but rather phenotypic variants of regular cells. Persisters play a major role in resistance of bacterial biofilms to death, and are likely to be responsible for recalcitrance of chronic infections to antibiotics. A lead into the mechanism by which these specialized survivor cells arise comes from the fact that DNA damage induces the SOS response in bacteria, a signaling pathway that up-regulates DNA repair functions. SOS response induction also leads to expression in *E. coli* of a *tisB* “toxin” gene encoding a small membrane-acting peptide that leads to a decrease in ATP and can kill cells if artificially overexpressed. We reasoned that *tisB* may actually be a persister gene and its product induces reversible dormancy by shutting down cell metabolism. We show that a knockout of *tisB* resulted in a sharply decreased frequency of persisters tolerant to ciprofloxacin, an antibiotic that causes DNA damage, whereas mild overproduction of the peptide induced persister formation. TisB-dependent persisters also were highly tolerant to unrelated antibiotics. It appears that production of persisters tolerant to all antimicrobials is a “side-effect” of fluoroquinolone antibiotics. Our results suggest that induction of TisB by the SOS response controls production of multidrug-tolerant cells and represents, to our knowledge, the first mechanism of persister formation.

## Introduction

Bacterial populations form *persisters*, dormant cells that are highly tolerant to antibiotics and play an important role in recalcitrance of biofilm infections [1],[2]. Time-dependent or dose-dependent killing by antibiotics is distinctly biphasic, revealing a surviving subpopulation of persister cells. Reinoculation of surviving cells produces a culture with a new subpopulation of persisters, showing that these cells are not mutants, but rather phenotypic variants of the wild type [3],[4]. Re-exposure of persisters to a different bactericidal antibiotic resulted in little or no additional killing, showing that persisters are multidrug-tolerant cells [5]. Gain-of-function mutants in the *E. coli hipA* toxin gene lead to an increase in the frequency of ampicillin- and fluoroquinolone-tolerant persisters in a growing population from 1 in 10,000 cells or less (wild-type levels) to 1 in 100 cells [6]–[10], and this *hipA7* mutant was shown to form persisters prior to addition of antibiotic [11]. These persisters were slow- or nongrowing cells. Wild-type persisters have been isolated from an exponential culture of *E. coli* untreated with antibiotic, by sorting out dim cells of a strain expressing a degradable GFP that is transcriptionally fused to a ribosomal RNA promoter [12]. This indicated that persisters are cells that have diminished protein synthesis and are dormant. The apparent dormancy of persisters accounts for their tolerance to bactericidal antibiotics whose action requires an active, functional target [13]–[16].

The mechanism of persister formation is currently unknown. Isolated persisters show increased expression levels of chromosomal toxin/antitoxin (TA) genes [9],[12]. Ectopic overproduction of RelE, an mRNA endonuclease [17], inhibits protein synthesis and creates dormant, multidrug-tolerant cells [9]. The HipA protein is an Ef-Tu kinase [18],[19], which also inhibits protein synthesis and produces multidrug-tolerant cells upon overproduction.

However, strains deleted in individual TA loci do not have a phenotype [9],[12], possibly due to their functional redundancy [20]–[22]. In *E. coli*, there are at least 15 TA modules [20],[22],[23]. Importantly, a screen of an ordered 3,985 open reading frame (out of a total of 4,288) knockout library of *E. coli* [24] for mutants lacking persisters in stationary phase produced a largely negative result—not a single strain lacking persister formation was identified [25]. Similar negative findings were reported with screens of *E. coli* transposon insertion (Tn) libraries [26],[27] and a *Pseudomonas aeruginosa* Tn library [28]. Only mutants with modest reduction in persister levels were identified, and in the case of *E. coli*, these were primarily in global regulators [25]. This strongly suggests that there are multiple, redundant mechanisms of persister formation. Persisters were originally described by Bigger in 1944 [3], but functional redundancy has made it very challenging to elucidate the mechanism by which they form.

A useful clue to a possible mechanism of persister formation comes from the analysis of the SOS response. Interestingly, SOS induces several TA genes in *E. coli*, whose promoters contain a Lex box: *symER*, *hokE*, *yafN/yafO*, and *tisAB/istR* [23],[29]–[35] Another locus, *dinJ/yafQ*, contains a Lex box but is not believed to be under SOS control [29],[30]. Importantly, only the toxin gene is predicted to be up-regulated in the three type 1 TA modules (*symER*, *hokE*, and *tisAB/istR*) following SOS induction, whereas in the type 2 TA modules, toxin and antitoxin form an operon and are therefore both expected to be induced. Fluoroquinolones such as ciprofloxacin induce the SOS response [36] by blocking the ligase activity of DNA gyrase and topoisomerase, converting them into endonucleases [14],[37]. In a separate study, we have shown that the SOS response is also necessary for persister formation in response to the fluoroquinolone antibiotic ciprofloxacin [38]. In the present study, we examine the mechanism of this ciprofloxacin-induced persister formation and find that it is governed by the TisB toxin.

## Results

**Figure 1:**
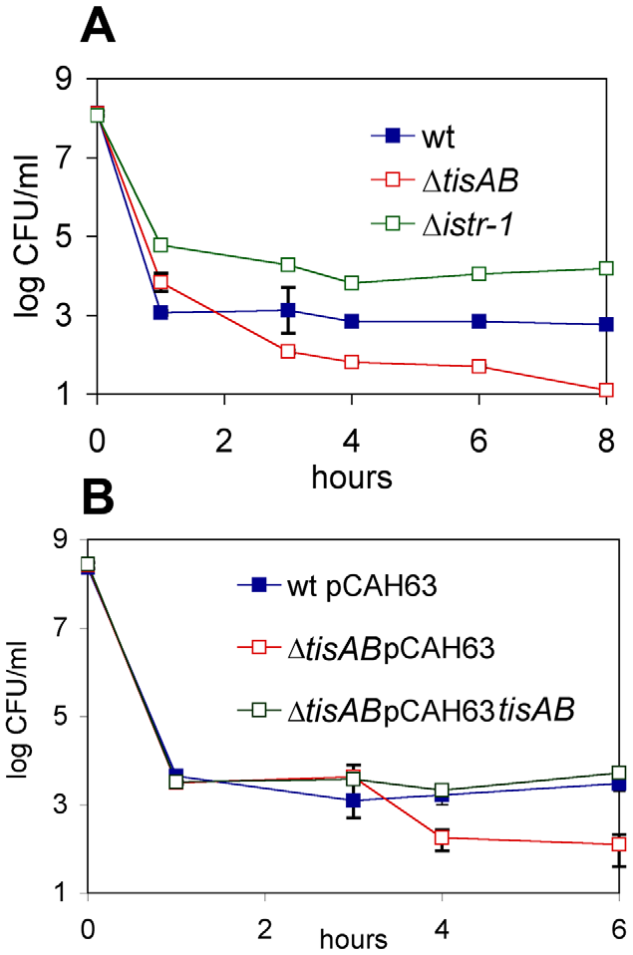
Survival of the *tisAB/istR* mutants after ciprofloxacin exposure and complementation of the phenotype. (A) Knockout strains of the toxin locus *tisAB* and its antitoxin *istR-1* were exposed to 1 μg/ml ciprofloxacin in exponential growth phase and survival determined by spot plating for colony forming units. The graph is a representative of at least five independent experiments with similar results, error bars indicate the standard error. (B) MG1655 Δ*tisAB* carrying the *tisAB* region as a single-copy insertion in the lambda attachment site was treated as described in (A). wt, wild type.

Ciprofloxacin rapidly killed the bulk of *E. coli* cells, leaving surviving persisters (Figure 1). Strains deleted in one of the five SOS-TA loci were examined for time-dependent killing by ciprofloxacin, and one of them, Δ*tisAB* (GenBank accession number NC_000913), had a sharply decreased level of persisters (Figure 1A). This suggests that the majority of persisters, ≥90%, were formed in response to ciprofloxacin treatment, and their production is dependent on *tisAB*. Introduction of *tisAB* in single copy into the lambda attachment site of the Δ*tisAB* strain complemented the low persister phenotype of the knockout strain (Figure 1B). Persister levels observed in time-dependent killing experiments with ampicillin or streptomycin that do not cause DNA damage were unchanged in the Δ*tisAB* strain (unpublished data). Ampicillin has been reported to induce the SOS response [39], but apparently the level of induction is insufficient to influence TisB-dependent persister formation.

**Figure 2:**
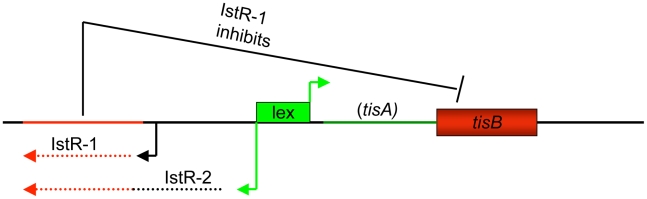
Schematic of the *tisAB/istR* locus. Only the LexA-controlled toxin *tisB* is translated in vivo; *tisA* contains the binding site for the constitutively expressed antitoxin RNA IstR-1 [36]. The IstR-2 RNA is under LexA control and contains the entire IstR-1 RNA. Its role in *tisAB* regulation is currently unclear.

IstR-1 is an antisense RNA antitoxin that is expressed constitutively from its own, LexA-independent promoter and controls the production of the TisB toxin [28]. IstR-2 is a longer small RNA transcript that is LexA controlled and contains the entire IstR-1 RNA sequence. IstR*-*2, however, has been suggested not to be involved in the control of TisB production [40]. *tisA* is an untranslated open reading frame that contains the antisense RNA binding site as well as the ribosome binding site for *tisB* [32]. A schematic of the *tisAB/istR* locus based on [40] is shown in Figure 2.

A strain deleted in *istR-1* caused a marked, 10- to 100-fold increase in the level of persisters (Figure 1A). This is consistent with increased levels of TisB leading to persister formation. This result is also in apparent contradiction to a published study showing that ectopic expression of *tisB* kills cells [41]. It seems likely that the high levels of expression from the multicopy plasmid used in the above-cited study were responsible for cell death. Importantly, the minimal inhibitory concentration (MIC) of ciprofloxacin for *tisAB* and *istR-1* knockouts was the same as in the wild type, showing that these genes do not affect resistance to this antibiotic, but rather control drug tolerance by modulating persister production. To test whether IstR-2 was also involved in *tisB* regulation in persisters, we produced a knockout of the *istR-2* promoter region and tested it for ciprofloxacin-induced persister formation. Unexpectedly, the ΔP*istR-2* strain had reduced persister levels similar to the *tisAB* knockout (Figure S1). It is possible that the *istR-2* promoter region contains a binding region of a positive regulator that is essential for *tisB* expression.

**Figure 3:**
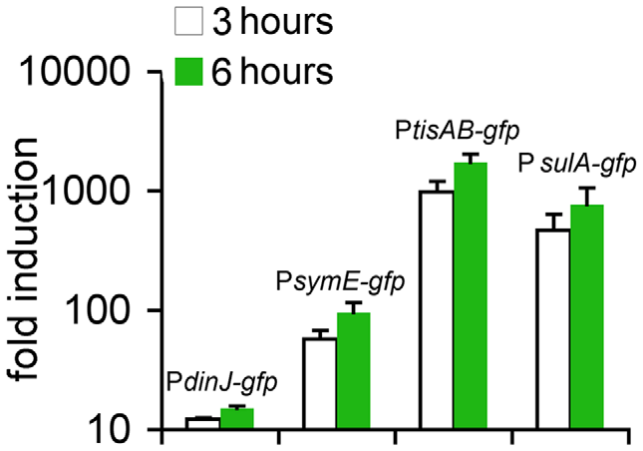
Induction of LexA-controlled promoters by ciprofloxacin. Cells carrying plasmid-borne promoter-*gfp* fusions were exposed to 0.1 µg/ml ciprofloxacin in exponential phase. Fold induction is GFP fluorescence after 3 h (open bars) and 6 h (green bars) of exposure normalized to initial fluorescence. This graph is a representative of three independent experiments with similar results; error bars indicate the standard error.

Using a plasmid-borne promoter-*gfp* fusion, we measured induction of *tisAB* in response to ciprofloxacin, and compared this to the expression of other SOS-TA genes (Figure 3). The *tisAB* promoter was the most active after 6 h of exposure to ciprofloxacin and showed a 1,000-fold induction, followed by the *symE* promoter, which showed a 100-fold induction. *tisAB* promoter activity was even higher than that of the *sulA* promoter, a standard readout of the SOS response. The *dinJ/yafQ* promoter was not significantly activated by ciprofloxacin. This is in agreement with a previous report showing that despite the presence of a putative LexA binding box, the *dinJ/yafQ* locus may not be under control of the SOS response [29]. The results of the induction experiment are consistent with the prominent role of TisB in persister formation in response to ciprofloxacin.

**Figure 4:**
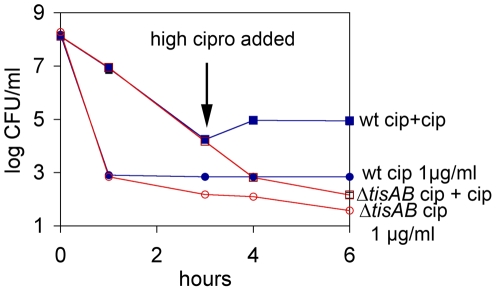
Adaptive ciprofloxacin tolerance in *E. coli*. Wild-type MG1655 and its Δ*tisAB* derivative were grown to exponential phase and exposed to 0.1 µg/ml ciprofloxacin (cipro/cip) for 3 h, after which 1 µg/ml ciprofloxacin was added (ciprofloxacin MIC is 0.016 µg/ml). As a control, a parallel culture was immediately exposed to 1 µg/ml. Viable cell number was determined by serial dilution and plating for colony forming units (CFU/ml). The data points are averages of three independent experiments; error bars indicate the standard error. wt, wild type.

A common feature of inducible responses is an increase in tolerance upon repeated exposure to a noxious factor. In a separate study [38], we showed that ciprofloxacin induces persister formation in a typical step-wise induction experiment (exposure to a low dose of an antibiotic followed by a higher dose). Here, we wanted to test whether *tisB* was responsible for this phenotype. Wild-type *E. coli* cells were pre-exposed to low levels of ciprofloxacin (0.1 µg/ml, 5×MIC) followed by a higher dose (1 µg/ml) of the same antibiotic (Figure 4). In a control experiment, the population was exposed to the high dose from the beginning. Step-wise exposure resulted in a 10- to 100-fold higher persister level as compared to a population that was immediately exposed to a high dose of the antibiotic. This pattern is typical of an adaptive response. In contrast to the wild type, pretreatment with a low dose of antibiotic did not induce a higher level of surviving persisters in the Δ*tisAB* mutant. This shows that this adaptive response to ciprofloxacin depends on *tisAB*.

**Figure 5:**
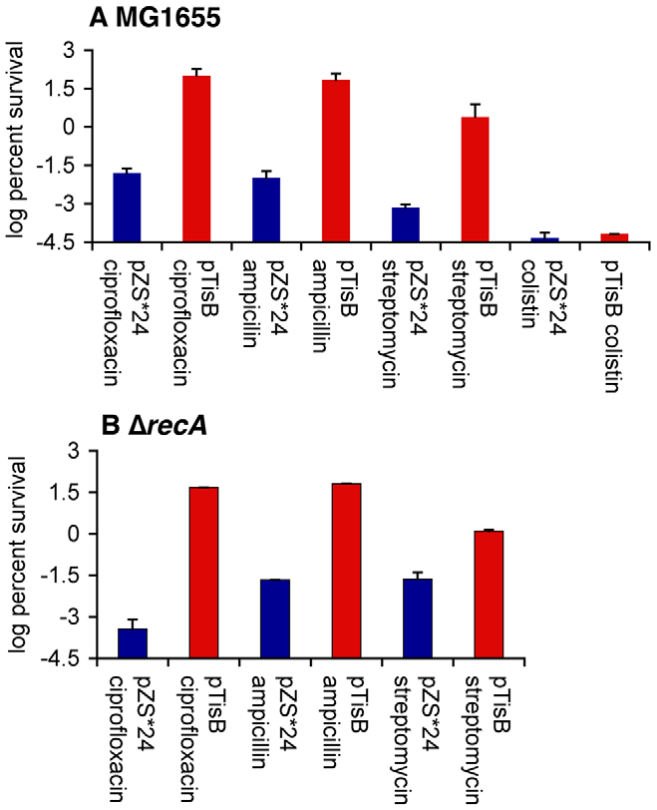
TisB overproduction and antibiotic tolerance. *tisB* was overexpressed in (A) MG1655 and (B) MG1655 Δ*recA* in exponential phase from a low copy number vector and exposed to ciprofloxacin (1 µg/ml), ampicillin (50 µg/ml), streptomycin (25 µg/ml), or colistin (10 µg/ml). Survival after 3 h was compared to a control strain carrying vector without *tisB*. The graph shows averages of three independent experiments; error bars indicate the standard error.

Next, we tested the ability of persisters formed in response to *tisB* expression to tolerate multiple antibiotics. For this purpose, *tisB* was cloned into a low-copy-number vector pZS*24 with an IPTG inducible promoter, and the toxin gene was expressed in exponentially growing cells. Growth leveled off approximately 1 h after the addition of IPTG (unpublished data). Cells overproducing TisB were exposed to antibiotics from four unrelated classes, and survival was measured after a 3-h incubation (Figure 5). As expected of nongrowing cells, the strain overproducing TisB was completely tolerant to ampicillin, a cell wall synthesis inhibitor that only kills growing cells. Interestingly, cells overproducing TisB were completely tolerant to ciprofloxacin as well. In contrast to ampicillin, ciprofloxacin is very effective in killing regular nongrowing cells, even those without ongoing replication [4],[9],[42]. It appears that TisB produces persisters highly tolerant to this DNA-damaging agent. TisB-producing cells also survived exposure to streptomycin, a protein synthesis inhibitor, 100-fold better than the control strain. This shows that TisB-dependent persisters exhibit multidrug tolerance. Antibiotics tested in these experiments act against defined targets. Decreased activity of the target functions in persisters would lead to drug tolerance. Persisters formed by TisB overproduction were susceptible to colistin, a polypeptide antibiotic permeabilizing the outer membrane [43]. This is expected, since an intact outer membrane is essential for cell survival. Further, TisB overproduction protected a Δ*recA* mutant against bactericidal antibiotics from three different classes (Figure 5B).

**Figure 6:**
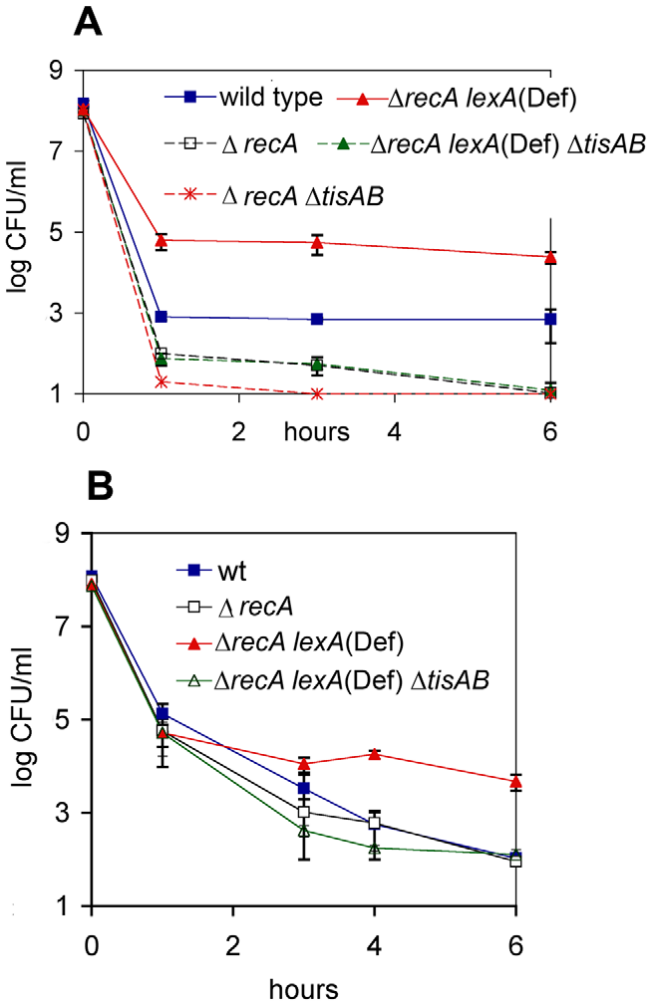
TisB-dependent persister formation in SOS response mutants. *E. coli* MG1655 and its derivatives Δ*recA*, Δ*recA* Δ*tisAB*, Δ*recA lexA*300(Def), and Δ*recA lexA*300(Def) Δ*tisAB* were grown to exponential phase and exposed to (A) ciprofloxacin at 1 µg/ml or (B) tobramycin at 20 µg/ml. Data are averages of at least three independent experiments; error bars indicate the standard error. wt, wild type.

The SOS response is initiated when RecA senses damaged DNA and activates cleavage of the global repressor LexA. It was important to establish whether TisB-dependent formation of persisters was controlled by this well-studied SOS response pathway. The persister level of a Δ*recA* strain treated with ciprofloxacin was lower as compared to the wild type, and similar to that of a Δ*recA* Δ*tisB* double mutant (Figure 6A).

*E. coli* can also constitutively express SOS-controlled genes if the LexA repressor is deleted. The level of surviving persisters in *E. coli* Δ*recA lexA*300(Def) treated with ciprofloxacin was dramatically increased as compared to the wild type (Figure 6A). Importantly, the MIC of the *E. coli* Δ*recA lexA*300(Def) to ciprofloxacin is 0.002, which is 8-fold lower than in the wild type. RecA is the main recombinase participating in DNA repair, which explains the increased susceptibility of the mutant to fluoroquinolones that cause double-strand breaks. This experiment clearly distinguishes between the decreased resistance of the regular cells, and increased levels of drug-tolerant persisters in the *E. coli* Δ*recA lexA*300(Def) population. Finally, we deleted the *tisAB* locus in Δ*recA lexA*300(Def) and measured survival in response to ciprofloxacin (Figure 6A) and tobramycin (Figure 6B). Persister levels in the Δ*tisAB* Δ*recA lexA*300(Def) triple mutant were drastically reduced as compared to the Δ*recA lexA*300(Def) strain and were similar to that of the Δ*recA* single deletion after exposure to either antibiotic.

**Figure 7:**
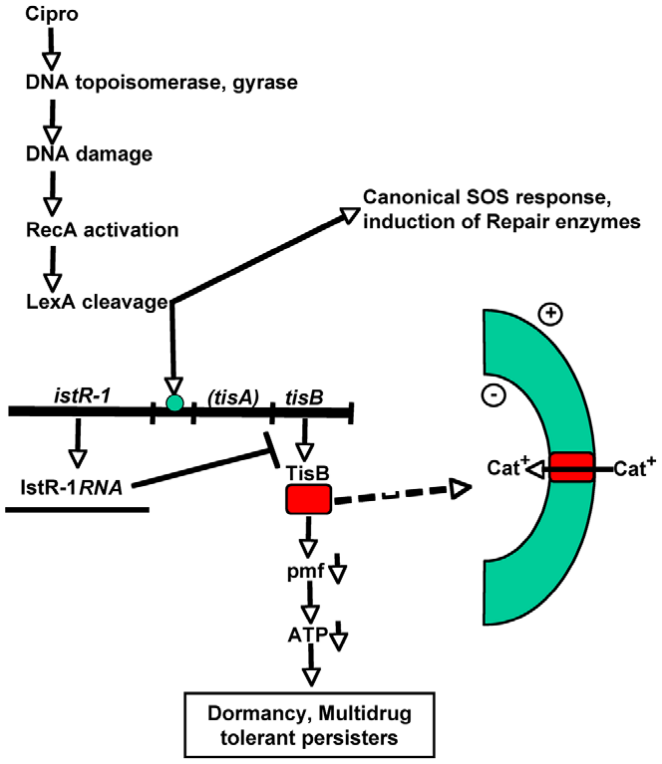
Model of ciprofloxacin-induced persister formation. Ciprofloxacin induces the SOS response, which up-regulates DNA repair functions. In a subpopulation of cells, the SOS response also induces the TisB toxin to a high level, which causes a decrease in proton motive force and ATP level, leading to multidrug tolerance.

Taken together, these experiments show that the SOS response triggers induction of TisB, causing formation of multidrug-tolerant persisters (Figure 7).

## Discussion

Previous research clearly indicated redundancy in persister formation mechanisms, suggesting a unique design of this cell-surviving function [2]. Indeed, all other complex systems of bacteria are made of components usually linked into a single linear pathway, and a screen of a knockout library readily identifies the genes. By contrast, a screen of a knockout library did not result in discovery of strains lacking persisters, and the only genes that were identified as contributing to the persister phenotype were global regulators (*hnr*, *dksA*, *fis*, *hns*) and genes involved in nucleotide metabolism (*apaH*, *yigB*) [25]. The screen was done in stationary phase, and the library did not contain a *tisAB* knockout strain. TisB-dependent persister formation is observed under conditions of maximal expression of the SOS response, which is in exponentially growing cells. Consistent with this, we did not observe a phenotype for the Δ*tisAB* strain in stationary phase (unpublished data), suggesting that under these conditions, persisters form through other mechanisms. The screen [25] did identify the upstream elements of *tisB* induction, *recA* and *recB*. These knockout strains have increased susceptibility to fluoroquinolones and were therefore initially not considered as valid candidates for persister genes.

Another persister component, the *glpR* regulon, was identified in a selection of an expression library of *E. coli* for increased drug tolerance [27]. Perhaps this redundancy of mechanisms evolved in response to antibiotics in the natural environment. If persisters are specialized survivors, then having multiple mechanisms of formation would ensure that no single compound will lead to their elimination.

This underscores the challenges in finding approaches to persister eradication. Redundancy of mechanisms is also challenging for identifying these mechanisms. Given that persisters are dormant, the search narrows for determinants that can reversibly block cellular functions. TA loci contain attractive candidates for persister genes. HipA encoded by the *hipBA* locus was the first candidate persister gene identified by a targeted selection for high-persister mutants [6],[7]. The *hipA7* allele carries a gain-of-function mutation that causes an increase in persister formation [4],[8]. Our recent studies showed that HipA is a protein kinase that phosphorylates EF-Tu, rendering it nonfunctional [18],[19]. Inhibition of protein synthesis leads to multidrug tolerance and presents a compelling scenario for persister formation. However, deletion of *hipBA* has no phenotype ([25]; an earlier report of a phenotype [9] was due to deleting a flanking region). Expression of other toxins (RelE; MazF [9],[44]) similarly leads to multidrug tolerance, but deletions do not have a phenotype. Extreme redundancy of TA genes would explain the lack of a phenotype, and therefore it seemed useful to search for conditions where a particular toxin would be expressed in a wild-type strain, and then examine a possible link to persister formation.

Several TA genes are expressed under conditions of the SOS response, which is induced by fluoroquinolone antibiotics. Examination of deletion strains showed that the level of persisters dropped dramatically in a Δ*tisAB* mutant and increased equally in a Δ*istR-1* mutant overproducing TisB. During steady-state growth, a fraction of cells induces the SOS response stochastically, which could have resulted in production of TisB-dependent persisters [45]. However, the level of persisters surviving treatment with streptomycin or ampicillin was not affected by the absence of *tisB*. This suggests that spontaneous SOS expression is insufficient to produce cells expressing enough TisB to cause dormancy. This is consistent with our findings that a strain unable to induce the SOS response exhibits reduced persistence in response to ciprofloxacin, but not ampicillin or streptomycin [38].

SOS caused by endogenous DNA damage during normal growth has been shown to induce a “viable but not culturable” state in a subpopulation of cells [45]. It is possible that this is the consequence of induction of SOS TA modules as well.

Ectopic overexpression of *tisB* sharply increased the level of persisters. Drug tolerance following artificial overexpression of a protein, however, may not be a good indicator of a bona fide persister gene. Ectopic overproduction of misfolded toxic proteins causing stasis produces an artificial state of drug tolerance in *E. coli* [44]. At the same time, overexpression experiments are necessary: if induction of a gene does not lead to an increase in drug tolerance, it can be safely eliminated as a candidate. Drop in persisters in a deletion strain and increase upon overexpression gives reasonable confidence in functionality of a persister gene. The dependence of TisB-induced persisters on a particular regulatory pathway, the SOS response, further strengthens the case for TisB as a specialized persister protein.

The long and unsuccessful search for a mechanism of persister formation has lead to the provocative hypothesis of dormant cells being formed by random fluctuations in any protein whose overproduction produces a toxic effect [44]. We previously showed that persisters are not formed in an early-exponential culture of *E. coli*, suggesting the presence of specific persister proteins, rather than random noise in expression of nonspecific genes [4]. However, this debate could only be settled with the identification of a persister protein. Our finding of an SOS-dependent induction of TisB resulting in multidrug tolerance suggests that there is in fact a specific mechanism of persister formation.

The role of TisB in persister formation is unexpected based on what we know about this type of proteins. TisB is a small, 29 amino acid hydrophobic peptide that binds to the membrane and disrupts the proton motive force (pmf), which leads to a drop in ATP levels [41]. Bacteria, plants, and animals all produce antimicrobial membrane-acting peptides [46]–[48]. Toxins of many TA loci found on plasmids belong to this type as well, and represent the plasmid maintenance mechanism. If a daughter cell does not inherit a plasmid, the concentration of a labile antitoxin decreases, and the toxin such as the membrane-acting *hok* kills the cell [49]. High-level artificial overexpression of *tisB* also causes cell death [41]. It is remarkable from this perspective that the membrane-acting TisB under conditions of natural expression has the exact opposite effect of protecting the cell from antibiotics. Cells expressing *tisB* stop growing, and the drop in pmf and ATP levels will shut down the targets of bactericidal antibiotics. Ciprofloxacin kills cells primarily by converting its target proteins, DNA topoisomerases, into DNA endonucleases [14],[50]. A drop in ATP will then prevent topoisomerases from damaging the DNA. β-lactams such as ampicillin kill by activating the autolysins [15],[51], and this requires active peptidoglycan synthesis by the target penicillin-binding proteins. Peptidoglycan synthesis ceases in nongrowing cells. Similarly, the aminoglycoside streptomycin requires an active ribosome for its killing action. Aminoglycosides kill primarily by interrupting translation, which creates toxic, misfolded peptides [13],[52]. Antibiotics also induce the formation of reactive oxygen species, which contributes to killing [16], and this requires an active target as well. By creating a dormant state, TisB causes a shutdown of antibiotic targets and multidrug tolerance. Fluoroquinolones such as ciprofloxacin are widely used broad-spectrum antibiotics, and their ability to induce multidrug-tolerant cells is unexpected and a cause of considerable concern. Induction of persister formation by fluoroquinolones may contribute to the ineffectiveness of antibiotics in eradicating biofilm infections. Indeed, pre-exposure with a low dose of ciprofloxacin drastically increases tolerance to subsequent exposure with a high dose [38].

Induction of persisters by the SOS-induced TisB toxin links together two seemingly opposite strategies of survival: active repair, and entry into a dormant state. It seems that in the presence of DNA-damaging factors, the optimal strategy is to both induce repair and increase the number of dormant cells, which will survive when everything else fails. Indeed, a progressive increase in the concentration of fluoroquinolones rapidly kills regular cells but has little effect on the survival of persisters ([53]; this study). This means that it is the dormant persisters rather than regular cells with induced repair that will ultimately survive the DNA-damaging antibiotic.

Apart from describing a key element of persister formation, this study also provides a precedent for a physiological function for a chromosomal TA gene pair. Although the role of TAs in plasmid maintenance is well established, the function of chromosomal TAs remains largely unknown. In a recent study, Van Melderen and coauthors produced a knockout of *E. coli* lacking five toxins, including the well-studied RelE and MazF (mRNA endonucleases) (Tsilibaris et al. [21]). The deletion strain had no apparent phenotype and showed normal growth, susceptibility to antibiotics, and stringent response. In *Erwinia chrysanthemi*, the chromosomal *ccdAB* TA module prevented postsegregational killing of cells that lost an F plasmid, which contains a homologous *ccdAB* locus [54]. Prevention of postsegregational killing may be a function of some TA genes but would not explain the presence of >80 TAs in the chromosome of *Mycobacterium tuberculosis* [55],[56], for example, which is not known to harbor plasmids. Induction of TA genes under specific conditions such as described in this study may shed some light on their function.

This study opens an intriguing possibility of a wider link between other stress responses and persister formation. Pathogens are exposed to many stress factors in the host environment apart from DNA-damaging agents, including oxidants, high temperature, low pH, membrane-acting agents. It is possible that all stress responses induce the formation of a small but resilient subpopulation of surviving persisters.

## Materials and Methods

### Media and Growth Conditions

Experiments were conducted in 0.1 M HEPES-buffered (pH 7.2) Mueller Hinton Broth (MHB) enriched with 10 mg/l MgSO_4_ and 20 mg/l CaCl_2_ according to NCCLS guidelines for susceptibility testing. Killing experiments were conducted by diluting overnight cultures 1∶100 in 3 ml of fresh medium in culture tubes, growing to approximately 2×10^8^ colony forming units (CFU)/ml and challenging with 0.1 or 1 µg/ml ciprofloxacin. For CFU counts, cells were plated on LB agar plates containing 20 mM MgSO_4_ to minimize carryover effects of ciprofloxacin.

### Strain Construction

Strains MG1655 Δ*tisAB*::FRT, Δ*IstR-1::*FRT, and ΔP*istR*-2::*cat* are precise deletions constructed using the method of Datsenko and Wanner [57] and cured of their chloramphenicol resistance cassette with pCP20 where applicable.

P1 transduction was used to move the delta *recA*::Kan, delta *sulA*::Kan alleles (from the MORI KEIO collection [24]) and *lexA*300(Def) (kindly provided by G. Walker) into the MG1655 background.

Strain MG1655 pZS*24*tisB* was constructed by cloning the *tisB* ORF into the Kpn1/Cla1 sites of pZS*24 [58] using primers tisBfwKpn1 (5′-GTAGTA**GGTACC**ATGAACCTGGTGGATATCGCCA**-**3′, Kpn1 site in bold) and tisBrevCla1 (5′ GTAGTA**ATCGAT**ACTTCAGGTATTTCAGAACAGCAT-3′, Cla1 site in bold).

MG1655 pUA66P*tisB-gfp* was constructed by cloning the *tisAB* promoter region into the XhoI/BamHI sites of vector pUA66*gfp* using primers PromTisFwXho1 (5′-GTAGTA**CTCGA**GGCCGGAGCGAGGTTTCGT-3′, Xho1 site in bold) and PromTisRevBamH1 (5′-GTAGTA**GGATCC**AACACAGTGTGCTCACGCGG-3′, BamH1 site in bold). The other promoter-*gfp* fusions were taken from a commercial library [59].

For complementation experiments, the *tisAB* locus was cloned into the CRIM vector pCAH63 using primers RegiontisBAfwKpn1 (5′-GTCGTC**GGTACC**TTGAGTATCGATCACAGTTTGCGT-3′, Kpn1 site in bold) and RegiontisBArevKpn1 (5′-GTCGTC**GGTACC**CCTTTGGTGCGACTTGAATCTG-3′, Kpn1 site in bold) and inserted into the lambda attachment site of strain MG1655 Δ*tisAB::FRT* as described by Haldimann and Wanner [60].

### Promoter Activity Assay

Cells carrying pUA66-promoter-*gfp* fusions were grown in MHB to exponential phase as stated before and exposed to ciprofloxacin. At each time point, aliquots were removed, washed 2×in 1% NaCl, and then transferred to a 96-well plate. GFP fluorescence was measured with Ex/Em 485/515 on a Gemini XS spectrophotometer (Molecular Devices). Induction was normalized to background (pUA66*gfp)*, CFU/ml, and initial fluorescence.

### *tisB* Overexpression and Persistence

MG1655 carrying either pZS*24 or pZS*tisB was grown to exponential phase in 12 ml of MHB in 125-ml baffled flasks containing 20 µg/ml kanamycin. TisB expression was induced for 2 h in mid-exponential phase by addition of 500 µM IPTG. The culture was then split and exposed to either ciprofloxacin (1 µg/ml), ampicillin (50 µg/ml), streptomycin (25 µg/ml), or colistin methane sulfonate (10 µg/ml) for 3 h.

## Supporting Information

Figure S1 **Persister formation in a strain with an** ***istR-2*** **promoter deletion.** Cells were grown to exponential phase and exposed to 0.1 µg/ml ciprofloxacin for 3 h to induce TisB, followed by a higher dose (1 µg/ml) for another 3 h. Cell survival was assessed by spot plating for colony forming units.
(0.12 MB TIF)

## Abbreviations

MIC: minimum inhibitory concentration
TA: toxin/antitoxin
