# Supplementary figures and images for: Ciprofloxacin Causes Persister Formation by Inducing the TisB toxin in Escherichia coli
Source: PLoS Biol. 2010 Feb 23;8(2):e1000317. doi: 10.1371/journal.pbio.1000317 (PMC2826370; doi:10.1371/journal.pbio.1000317)

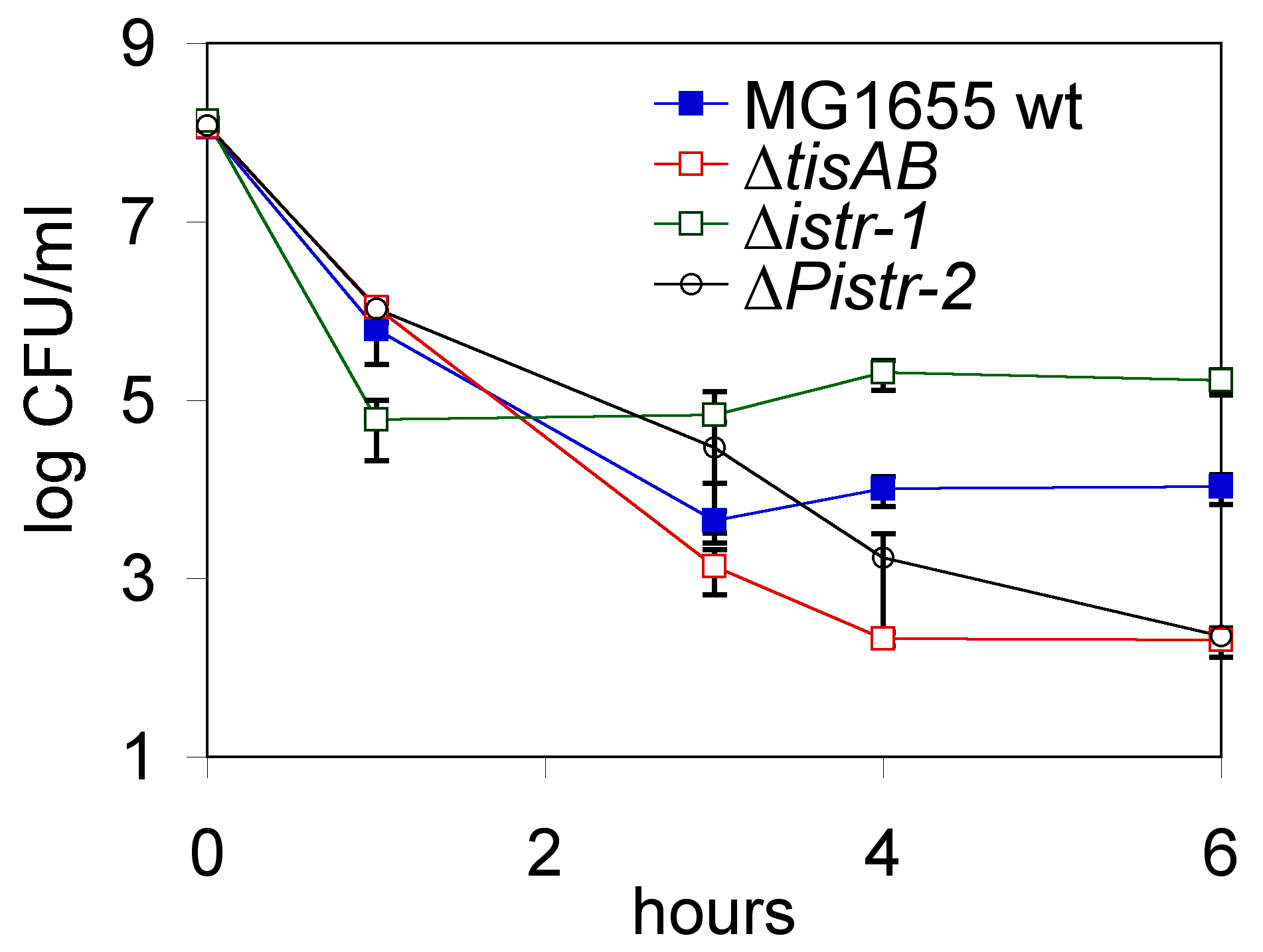

Supplement: Figure S1 — Persister formation in a strain with an istR-2 promoter deletion. Cells were grown to exponential phase and exposed to 0.1 µg/ml ciprofloxacin for 3 h to induce TisB, followed by a higher dose (1 µg/ml) for another 3 h. Cell survival was assessed by spot plating for colony forming units. (0.12 MB TIF) [file pbio.1000317.s001.tif]
